# Combined Resistance and Stretching Exercise Training Benefits Stair Descent Biomechanics in Older Adults

**DOI:** 10.3389/fphys.2019.00873

**Published:** 2019-07-17

**Authors:** James P. Gavin, Neil D. Reeves, David A. Jones, Mike Roys, John G. Buckley, Vasilios Baltzopoulos, Constantinos N. Maganaris

**Affiliations:** ^1^School of Health Sciences, University of Southampton, Southampton, United Kingdom; ^2^Research Centre for Musculoskeletal Science and Sports Medicine, Department of Life Sciences, Manchester Metropolitan University, Manchester, United Kingdom; ^3^Rise and Going Consultancy, Watford, United Kingdom; ^4^Department of Biomedical and Electronics Engineering, University of Bradford, Bradford, United Kingdom; ^5^Research Institute for Sport and Exercise Sciences, Liverpool John Moores University, Liverpool, United Kingdom

**Keywords:** stair negotiation, joint moments, stability, stretching, aging, movement control

## Abstract

**Introduction:**

Stair descent is a physically demanding activity of daily life and common risk for falls. Age-related deteriorations in ankle joint capacities make stair descent particularly challenging for older adults in built environments, where larger rise steps are encountered. Exercise training may allow older adults to safely cope with the high biomechanical demands of stair descent. However, little is known about the demands of increased rise stairs for older adults, nor the impact of exercise.

**Aim:**

We investigated whether the effects of lower-limb resistance training would alter joint kinetics and movement strategies for older adults when descending standard rise, and increased rise stairs.

**Methods:**

Fifteen older adults descended a four-step stair adjusted to standard rise (170 mm), and increased rise (255 mm) on separate visits. Between these two visits, randomly allocated participants underwent 16 weeks of either: resistance exercise training (*n* = 8) or habitual activity (*n* = 7). Kinetic data were measured from step-mounted force plates, and kinematic data from motion-capture cameras. Training involved twice-weekly sessions of lower-limb resistance exercises (three sets of ∼8 repetitions at ∼80% three-repetition maximum), and static plantarflexor stretching (three, 45 s holds per leg).

**Results:**

*Standard stairs* – Peak ankle joint moments increased (*p* < 0.002) and knee joint moments decreased (*p* < 0.01) during descent after exercise training. Peak centre of pressure-centre of mass (CoP-CoM) separations increased in posterior (*p* = 0.005) and medio-lateral directions (*p* = 0.04) after exercise training. Exercise training did not affect CoM descent velocity or acceleration. *Increased rise stairs* – Required greater ankle, knee, and hip moments (*p* < 0.001), peak downward CoM velocity and acceleration (*p* = 0.0001), and anterior-posterior CoP-CoM separation (*p* = 0.0001), but lower medial-lateral CoP-CoM separation (*p* < 0.05), when compared to standard stair descent. Exercise training did not affect joint kinetics or movement strategies.

**Discussion:**

Exercise training increased the maximum joint ROM, strength and force production of the ankle, and enabled a greater ankle joint moment to be produced in single-leg support (lowering phase) during standard stair descent. Descending increased rise stairs raised the task demand; exercise training could not overcome this. Future research should prioritize the ankle joint in stair descent, particularly targeting plantarflexor torque development across stairs of varying riser heights.

## Introduction

Stair negotiation is a challenging activity of daily living that people perform in both home and public environments. Stair descent presents a particular challenge, in that the individual must control the lowering of body mass in single-limb support, whilst the contralateral limb moves to the step below. This task involves lower limb joint ranges of motion and moments exceeding those required for level over-ground gait (McFadyen and Winter, 1988; Riener et al., 2002), and presents significant challenge to dynamic balance (Zachazewski et al., 1993). Adequate neuromuscular control is required to ensure that the swinging advancing foot negotiates the step edge bearing the loaded limb, as it moves forward and downward, and is then safely placed on the lower step. Muscular control at the ankle joint becomes critically important during landing as the ground reaction forces produced on step contact are dissipated in the landing-limb (Riener et al., 2002; Reeves et al., 2008a; Buckley et al., 2013). Muscle-tendon units undergo eccentric contractions in stair descent to decelerate body segments and absorb mechanical work. These further increase task demand for older people, who operate closer to their limits of eccentric ankle strength and dorsiflexion range, in comparison to younger people (Reeves et al., 2008a). As a coping strategy to meet this increased demand, older adults appear to redistribute joint moments, by maintaining knee joint moment (around 42% maximal), whilst lowering ankle joint moment within safer ranges (around 75% maximal) (Reeves et al., 2009), to operate within maximal capacities and reduce potential falls risk.

Falls incidence increases with age and is influenced by involvement in demanding daily activities. For example, 50% of adults aged 65 years and over, and 80% of adults aged 80 years and over, fall on average once a year (American Geriatrics Society et al., 2001), with the majority of falls occurring on stairs (Svanstrom, 1974; Hemenway et al., 1994). Previous work documenting age-related adaptations during stair descent have revealed valuable insights into potential biomechanical factors contributing to an increased falls risk in older adults (Buckley et al., 2013; Novak et al., 2016; Dixon et al., 2018). Older people operate at higher maximum eccentric ankle capacities and joint ranges than the young (Reeves et al., 2008a), which leaves little reserve capacity for the old to cope with unanticipated perturbations occurring in stair descent.

To lower the centre of mass (CoM) to the step below, the lower-limb eccentrically flexes under the control exerted by the joint moments produced. If the joint moments are not sufficiently high, then the lowering velocity may become too excessive. Older adults adopt conservative strategies to safely negotiate these demands in stair descent, notably reducing peak CoM and advancing limb downward velocities (Buckley et al., 2013). Age-related deterioration in eccentric ankle force-generating capacity would reduce the ability of older people to absorb an increased downward velocity (momentum) during landing.

Exercise training interventions may be important in supporting older adults to meet the biomechanical demands of stair descent. Resistance exercise training has been shown to improve maximal lower-limb function and mobility (Bean et al., 2004) and stair negotiation performance (Capodaglio et al., 2005) of healthy adults aged over 70 years. For overground walking, lower-limb stretching training appears effective in increasing hip extension motion and gait stride length after 10 weeks (Watt et al., 2011), whilst ankle plantarflexor stretching has been shown to increase range of motion, step length and step velocity after 4 weeks (Cristopoliski et al., 2009). However, even long-term (i.e., 12 months) combined resistance and aerobic exercise training may have scant impact on the gait speed and joint motions of older adults in stair descent (Mian et al., 2007), despite improvements in lower-limb muscle mass, strength, and power (Morse et al., 2005). Where others have combined exercises, none have adopted training programs that are targeted to the impaired muscle groups of the lower-limbs (e.g., ankle joint motion and strength), nor assessed the joint kinetics or movement strategies of older adults when descending stairs. A specific, combined strengthening (to better cope with the high joint moment demand) and stretching (to better cope with the high ankle dorsiflexion demand) program for the ankle and knee muscle groups is therefore necessary.

Potential adaptations conferred by exercise interventions to how stairs are negotiated should be studied across different stair-riser heights, as stairs and steps encountered in daily life can vary from those that are within national regulatory guidelines [e.g., maximum permitted stair riser for individual dwellings in the United Kingdom is 220 mm; (HM Government, 2013)], to those that are higher, as is the case for low-floor public transport vehicles (Institute for Transportation, and Development Policy [ITDP], 2018) and unregulated stairs and steps.

Even for young adults using the traditional step-over-step gait pattern, descending steps with risers increased by 50% (i.e., 255 mm) increases peak ankle (28.6%) and knee joint moments (29.8%), when compared to descending standard rise stairs (i.e., 170 mm) (Spanjaard et al., 2008). Descending stairs with increased rise increases joint moments and the challenge to balance, as individuals must step downward over greater vertical distance, and consequently generate greater joint moments to dissipate ground reaction forces and arrest CoM downward velocity. When adopting the step-over-step strategy for increased rise stair descent, older adults place greater demand on the ankle plantarflexors, whilst reducing knee extensor demand during landing (King et al., 2018). This presents further falls risk given older adults descend stairs closer to their ankle joint biomechanical limits. The biomechanical demands of stair descent are not known for older adults on increased rise steps, nor are the potential effects of exercise training. This is important as older adults operate closer to their maximal capacities, than the young.

It seems then appropriate to (i) assess whether exercise training can affect the locomotion of older adults when descending standard stairs, (ii) quantify the demands of descending increased rise stairs for older adults, and finally (iii) assess whether exercise training can reduce any additional demands presented by descending increased rise stairs in older adults. The main aims for the present study were divided to focus on standard stairs and increased riser stairs as follows:

(1) To investigate whether 16 weeks of lower-limb resistance and stretching exercise training would lead to an alteration in joint kinetics, and movement strategies in older adults when descending standard stairs.

(2) To determine whether descending stairs of increased riser height modifies joint kinetics and movement strategies in older adults, and whether exercise training can alter these biomechanics.

## Materials and Methods

### Participants

Fifteen older adults (eleven women, four men; mean ± SD; age, 75 ± 3 years; height, 1.62 ± 0.07 m; body mass, 69.3 ± 11.1 kg) from the local community provided written informed consent to participate in the study. Participants were then randomly allocated to exercise and control groups. The research protocol was approved of by the ethics committee of the Manchester Metropolitan University. All participants were free from recent musculoskeletal and neuromuscular injury that would influence gait.

### Experimental Protocol

Stair descent trials were performed on a four-step stair, with steps of 280 mm going (tread) and 900 mm width and adjustable to standard rise (170 mm), and 50% increased rise (255 mm). For reference, the top step below the landing was labeled as step one, and the floor at the stair base as step four (left limb contacting steps two and four; right limb contacting steps one and three). Eight participants (age, 75 ± 4 years; height, 1.62 ± 0.09 m; body mass, 69.7 ± 12.3 kg) negotiated standard rise and increased rise stairs in a counter-balanced order, on separate visits, before and after 16 weeks of resistance exercise and stretching training (twice weekly). A non-training, control group consisting of seven participants (age, 75 ± 2 years; height, 1.63 ± 0.06 m; body mass, 71.2 ± 11.5 kg) underwent stair testing before, and after the same period of time as the exercise intervention (control period), whilst continuing with their habitual activity. Random allocation was used to assign participants to the respective groups. Three trials were performed on each visit.

On each visit, participants walked down stairs bare-feet. Following familiarization trials, participants stood at the top of the stairs and, leading with the right leg, they were asked to walk down the stairs unaided, at a self-selected pace, in a step-over-step manner. Kinematic and kinetic analysis focussed on a single gait cycle for the left limb in steady-state, from the first touch-down onto step one, to the second touch-down onto step four (force plate on the floor), and subsequently averaged across the three trials. Gait cycles refer to the left leg, defined by the events of: initial foot contact (step two), single-leg stance (step two), double support, foot off (step two), and final foot contact with the step below (step four) (Nadeau et al., 2003; Reeves et al., 2008a). Single support represents the proportion of the gait cycle when the participant is supported by the left leg; double support represents support by both legs.

### Kinetic and Kinematic Data

Three piezoelectric force plates were mounted in each step (Kistler type Z17068, Kistler Instruments, Winterthur, Switzerland); the steel steps were bolted individually to the laboratory floor. One additional force plate was mounted in the concrete floor at the bottom of the stair (Kistler type 9253A, Kistler Instruments, Winterthur, Switzerland). Kinetic data were collected at 1,080 Hz, down-sampled to 120 Hz, and subsequently analyzed in anterior-posterior (sagittal plane), and medial-lateral (frontal plane) directions. Ground reaction forces were measured independently for each plate.

Nine motion-capture cameras (VICON 612 system, VICON Motion Systems Ltd., Oxford, United Kingdom) recorded the displacement of retro-reflective markers whilst the participants performed stair descent trials. Thirty-four markers were placed onto anatomical landmarks as recommended by the Helen Hayes plug-in-gait marker set. Markers were secured with double-sided, adhesive tape to the skin, or to tight-fitting shorts and t-shirt. Segmental motion data were sampled at 120 Hz. Captured descent trials were processed using Workstation software using participant anthropometric measures and the “plug-in-gait” model (VICON Motion Systems Ltd., Oxford, United Kingdom). For processing, gap filling was applied to marker trajectories with less than 10 missing samples; for 10 or more, trials were excluded. A Woltring filter was then applied (mean square error value, 20) to ensure constant treatment across the data-set. Finally, joint kinematics and kinetics were processed by running inverse kinematics and inverse dynamics analyses (static and dynamic Plug-in-Gait model) in the Workstation software. Kinematic and kinetic data for the ankle, knee, and hip joints, alongside toe, heel and CoM co-ordinates (*x*, *y*, *z*), were exported to ASCII format for further analysis. Data were analyzed in the sagittal plane for the ankle, knee and hip joints, and in the frontal plane for the hip joint according to previous observations (Nadeau et al., 2003; Mian et al., 2007).

### Maximum Functional Capacities

Eccentric, maximum voluntary contractions of the knee extensors and plantarflexors were assessed at baseline and 16 weeks later, for exercise training and control groups using an isokinetic dynamometer (Cybex NORM, New York, NY, United States). Using the left leg, participants performed three maximum contractions each (with 2 to 3 min rest), at angular velocities of: 60, 120, 180, and 240°⋅s^–1^. Knee extensions were performed seated, with the hip at 85° (hip supine = 0°); ankle plantarflexions were performed lying prone, with the knee at 0° (full extension) (Reeves et al., 2008a). Isometric, maximum voluntary contractions of the plantarflexors were assessed to determine the rate of torque development (Reeves et al., 2003); instruction was given to perform each contraction (lasting approximately 1 to 2 s) as rapidly as possible (Aagaard et al., 2002).

Maximum assisted dorsiflexion angle was also determined in the prone position with the knee extended to assess the impact of the stretching intervention on maximum dorsiflexion range of motion. With the left foot attached to the footplate, the investigator manually dorsiflexed the foot until the participant expressed they could no longer tolerate any further dorsiflexion movement.

### Joint Moment Normalization

Ankle and knee joint moments produced in stair descent were normalized relative to maximum capacities for each participant, as follows: (i) maximal eccentric extensor moments were quantified from the left leg across four different angular velocities (see section “Maximum functional capacities” above), (ii) the maximum joint moment in the gait cycle (i.e., the point of highest demand) and it’s corresponding angular velocity were then identified for each respective joint, and (iii) the angular velocity at maximum moment for gait was matched, to the most closely corresponding angular velocity for dynamometry-based measurements (Reeves et al., 2008a). Finally, the maximum moment in the gait cycle was divided by the dynamometry-based maximum moment for the corresponding angular velocity.

Joint power was determined for each lower limb joint throughout the gait cycle (unpublished data), as the product of joint moment and joint angular velocity. Joint mechanical work was computed by integrating the joint power curves of the ankle, knee and hip joints, respectively, using the trapezium method. Joint work was expressed as positive or negative to indicate when a joint was operating eccentrically (i.e., negative work) or concentrically (i.e., positive work). Positive work indicates energy production, whereas negative work indicates energy absorption.

### Center of Mass Calculations: Displacement, Velocity, and Acceleration

The centre of pressure-centre of mass (CoP-CoM) separation was characterized as the difference between the projections of the CoP of the ground reaction force vector (*x*, *y*, *z*), and CoM, for both sagittal and frontal planes. For trials where a participant had one foot on two separate force plates, a weighted average was calculated for CoP (Reeves et al., 2008b). Minimum and maximum values of CoP-CoM separation were used to represent anterior, and posterior separation in the sagittal plane, and medial and lateral in the frontal plane during each gait cycle.

Center of mass velocity and acceleration were determined following previous methods (Buckley et al., 2013), for the initial step down (i.e., transition from stair landing to step one) divided into descent and landing phases. Movement initiation was determined from when the vertical velocity of the lead limb’s heel marker first exceeded 0.05 m/s in the upward direction, for six consecutive frames (sampling rate, 120 Hz; time span, 0.01 s), whereas the subsequent foot contact (on to step one) was identified as when the vertical ground reaction force exceeded 20 N (descent phase). The landing phase was from foot contact, to the point at which downward CoM velocity reduced to zero or became positive. The following characteristics were identified for the initial step down: peak foot (i.e., heel) velocity, CoM peak downward velocity, CoM peak acceleration (descent phase), and CoM peak acceleration (landing phase).

### Resistance and Stretching Exercise Training

The participants undergoing exercise training performed small group sessions involving supervised resistance and stretching exercises for the lower-extremities twice a week, for 16 weeks. Resistance exercises were conducted on leg-press, knee extension and calf-press machines, with the three-repetition maximum (3RM) determined during session one. A standardized warm up (15 repetitions at 40% 3RM) commenced each exercise, which, following a short rest, involved three sets of ∼8 repetitions (75 to 80% 3RM). For the calf-press, three sets of 10 to 12 maximal, isometric contractions (1 to 2 s duration each, performed as rapidly as possible) were also performed to improve plantarflexor rate of torque development. The 3RM was reassessed every 4 weeks to monitor progression of exercise training load (Welle et al., 1996; Mian et al., 2007).

Plantarflexor muscles underwent static stretching, one leg at a time, prior to resistance exercises. Standing with the stretched leg extended on wedges inclined at 15 or 25° (Han et al., 2014). Stretches were held for 45 s, with three repetitions per leg. Intensity was raised by: (1) maintaining the stretched leg in extension, whilst shifting the supporting leg forward; and/or (2) increasing wedge incline from 15 to 25°. Stretching was performed to increase maximum dorsiflexion angle, because stair descent requires application of large dorsiflexion support moments, at a large dorsiflexion angle (Reeves et al., 2008a), far exceeding those during stair ascent (Protopapadaki et al., 2007; Reeves et al., 2009).

### Statistical Analysis

Two-way, mixed model ANOVAs [time (pre, post 16 weeks) (within-subject factor) × group (exercise, control) (between-subject factor)] were performed separately for standard and increased riser stairs to test the effects of (i) exercise training and (ii) step rise height, on the temporal-spatial characteristics, joint moments, and CoM parameters during stair descent at 0 and 16 weeks. Bonferroni adjustments were used to identify specific training effects for each group. Data analysis was performed using IBM SPSS Statistics Version 21 (IBM Corp, Armonk, NY, United States), with statistical significance accepted as *p* < 0.05.

## Results

### Maximum Functional Capabilities

Maximum eccentric knee torques assessed on a dynamometer increased at all angular velocities after exercise training (Table 1), whereas maximum eccentric ankle torque increased only at 60°⋅s^–1^. The angles of peak torque were not different for knee and ankle joints after exercise training (*p* > 0.05). For range of motion, maximum assisted dorsiflexion angle increased by 10.8% after exercise training (*p* = 0.03). Maximum isometric, rate of torque development increased after exercise training for both joints (knee 47.8%; ankle 21.7%; *p* < 0.05).

**TABLE 1:** Maximum functional capabilities of resistance exercising and non-exercising control older adults.

		**Exercise training group (*n* = 8)**		**Control group (*n* = 7)**	
			
	**Angular velocity (°⋅s^–1^)**	**Pre**	**Post**	**Pre**	**Post**
Maximum knee peak torque (N⋅m)	240	52.9 ± 28.5	72.4 ± 35.3^**^	70.2 ± 27.6	74.9 ± 31.1
	180	64.8 ± 38.6	92.8 ± 36.6^**^	94.5 ± 42.4	104.2 ± 25.1
	120	78.5 ± 38.3	106.0 ± 42.6^*^	104.1 ± 41.3	120.1 ± 22.0
	60	88.2 ± 35.8	114.4 ± 43.1^**^	117.1 ± 30.6	123.6 ± 32.0
Maximum ankle peak torque (N⋅m)					
	240	52.7 ± 14.4	58.8 ± 28.0	60.6 ± 22.5	56.8 ± 18.8
	180	71.9 ± 32.6	79.1 ± 31.5	82.3 ± 22.9	81.0 ± 26.7
	120	79.3 ± 36.6	103.9 ± 38.9	104.2 ± 29.7	100.0 ± 26.8
	60	86.3 ± 32.5	116.7 ± 35.0^**^	125.6 ± 37.1	108.1 ± 22.5^*^
Rate of knee torque development (N⋅m⋅s^–1^)		478.0 ± 255.4	706.4 ± 244.5^*^	407.3 ± 225.9	310.1 ± 240.3
Rate of ankle torque development (N⋅m⋅s^–1^)		324.6 ± 142.5	395.2 ± 131.5^*^	270.5 ± 98.3	327.1 ± 88.8
Maximum assisted dorsiflexion angle (°)		33.3 ± 4.3	36.9 ± 3.1^*^	33.6 ± 1.8	35.6 ± 3.1

Values are mean ± SD. Significant pre-post training difference, ^*^p < 0.05, ^∗∗^*p* < 0.01. Data correspond to the right limb.

### Standard Rise Stair Descent

#### Temporal-Spatial Characteristics

No accidents (e.g., slips, oversteps, or falls) occurred during descent trials of standard or increased rise stairs. Resistance exercise training did not affect gait characteristics during standard stair descent (stride frequency: 79.9 ± 15.6 steps/min, step length: 0.35 ± 0.03 m; single support duration: 46.0 ± 5.5%; double support duration: 25.6 ± 6.8%).

#### Joint Moments (Absolute)

**FIGURE 1:**
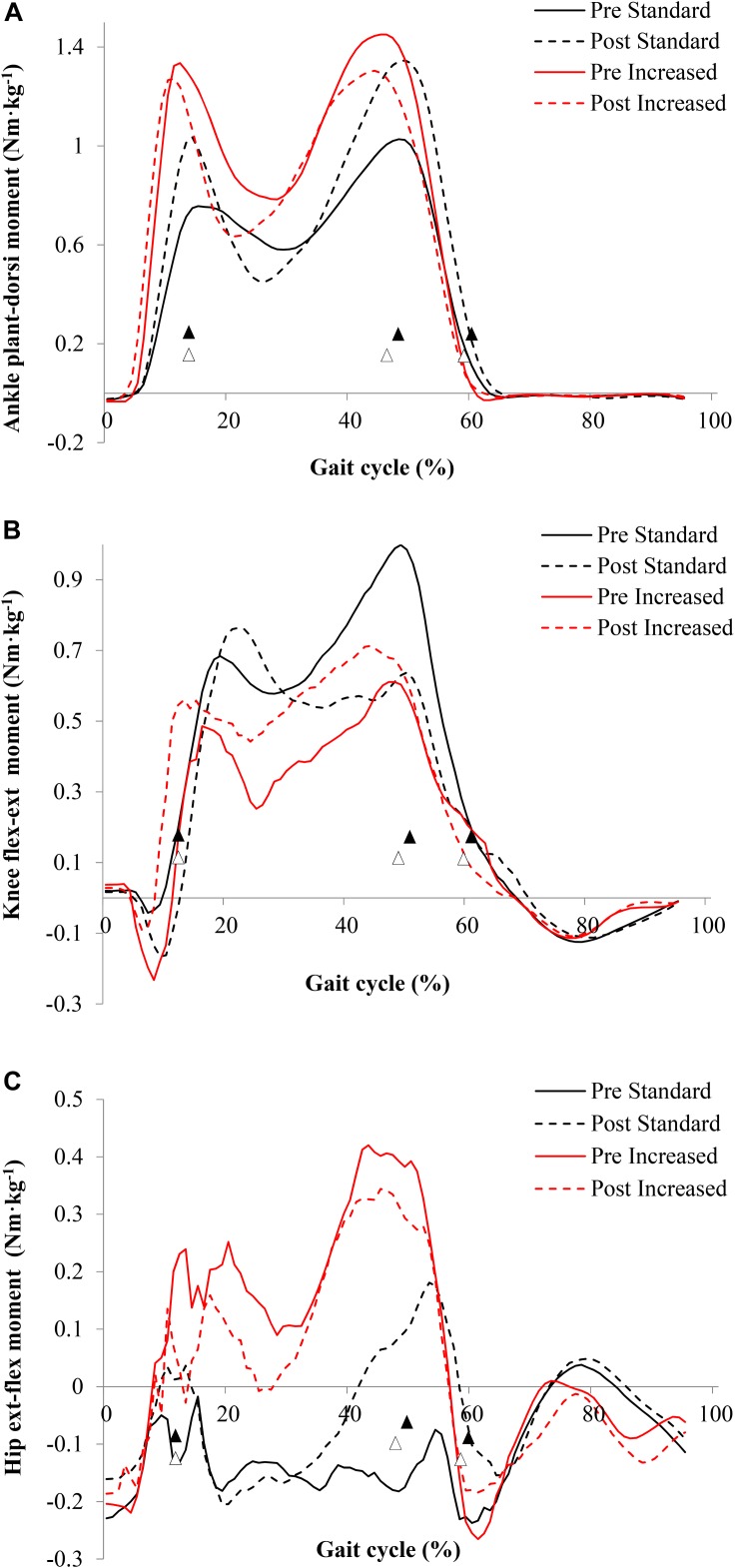
Peak joint moments of older adults descending stairs of different dimensions before and after 16 weeks of exercise training for the ankle **(A)**, knee **(B)**, and hip **(C)** joints. Gait cycle events (▲ standard, △ increased) refer to initial foot contact (0%), single-leg stance, double support, foot off, and final foot contact with the below step (100%); data correspond to the left limb. Values are mean (*n* = 8).

Peak ankle joint moments during both the single-leg support (lowering phase) (*F*_2,8_ = 6.25, *p* = 0.02, *d* = 0.33) and double support (landing phase) (*F*_2,8_ = 6.1, *p* = 0.027, *d* = 0.32) increased after exercise training (Figure 1 and Table 2). Peak knee joint extension moment decreased after exercise training (from the lowering phase) (*F*_2,8_ = 28.8, *p* < 0.01, *d* = 0.69; Table 3). Hip joint flexion moment peaks (during the swing phase, from single-leg stance to final left foot off; *p* < 0.05) increased in the swinging limb after exercise training.

**TABLE 2:** Ankle joint moments of older adults descending stairs of different dimensions before and after 16 weeks of exercise training.

	**Exercise group (*n* = 8)**		**Control group (*n* = 7)**		
			
**Gait cycle events**	**Pre**	**Post**	**Pre**	**Post**	***p*-value**
**Standard stairs**					
Initial foot contact (0%)	−0.03 ± 0.01	−0.02 ± 0.01	−0.02 ± 0.001	−0.02 ± −0.01	0.19
Single-leg stance	0.65 ± 0.14	0.97 ± 0.25^*^	0.67 ± 0.19	0.86 ± 0.33	0.02
Double support	1.02 ± 0.20	1.35 ± 0.26^*^	1.04 ± 0.10	1.16 ± 0.16	0.027
Foot off	0.07 ± 0.16	0.13 ± 0.24	0.09 ± 0.07	0.12 ± 0.19	0.65
Final foot contact (100%)	−0.01 ± 0.01	−0.02 ± 0.00	−0.025 ± 0.02	0.11 ± 0.30	0.25
Normalized maximum ankle moment (%)	0.89 ± 0.17	1.39 ± 0.63^*^	0.91 ± 0.35	1.06 ± 0.18	0.006
**Increased rise stairs**					
Initial foot contact (0%)	−0.03 ± 0.02	−0.03 ± 0.01	−0.03 ± 0.001	−0.03 ± 0.001	0.21
Single-leg stance	1.34 ± 0.34^†^	1.32 ± 0.39	1.45 ± 0.57	1.25 ± 0.35	0.44
Double support	1.35 ± 0.22†	1.13 ± 0.29^*^	1.42 ± 0.23	1.33 ± 0.19	0.021
Foot off	−0.01 ± 0.03^†^	0.01 ± 0.07	0.09 ± 0.16	0.08 ± 0.16	0.95
Final foot contact (100%)	−0.02 ± 0.01	−0.01 ± 0.01	−0.014 ± 0.001	−0.02 ± 0.03	0.18
Normalized maximum ankle moment (%)	1.75 ± 0.59^†^	1.78 ± 0.51	1.41 ± 0.58	1.54 ± 0.42	0.58

Values are mean ± SD. Significant difference: training ^*^, step dimension ^†^*p* < 0.05.

**TABLE 3:** Knee joint moments of older adults descending stairs of different dimensions before and after 16 weeks of exercise training.

	**Exercise group (*n* = 8)**		**Control group (*n* = 7)**		
			
**Gait cycle events**	**Pre**	**Post**	**Pre**	**Post**	***p*-value**
**Standard stairs**					
Initial foot contact (0%)	0.02 ± 0.03	0.02 ± 0.02	−0.01 ± 0.04	−0.004 ± 0.02	0.34
Single-leg stance	0.20 ± 0.1	−0.03 ± 0.34^*^	0.09 ± 0.10	−0.08 ± 0.22	0.02
Double support	1.00 ± 0.17	0.63 ± 0.25^*^	0.75 ± 0.19	1.07 ± 0.29	0.0001
Foot off	0.18 ± 0.12	0.17 ± 0.05	0.28 ± 0.10	0.24 ± 0.19	0.78
Final foot contact (100%)	−0.02 ± 0.03	−0.02 ± 0.03	−0.02 ± 0.04	−0.05 ± 0.04	0.68
Normalized maximum knee moment (%)	1.03 ± 0.48	0.67 ± 0.26^*^	0.75 ± 0.25	0.66 ± 0.26	0.01
**Increased rise stairs**					
Initial foot contact (0%)	0.04 ± 0.03	0.03 ± 0.03	0.01 ± 0.03	−0.01 ± 0.01	0.76
Single-leg stance	0.16 ± 0.41^†^	0.66 ± 0.37^*^	0.28 ± 0.40	0.33 ± 0.25	0.06
Double support	0.60 ± 0.24^†^	0.65 ± 0.29	0.50 ± 0.23	0.52 ± 0.24	0.82
Foot off	0.19 ± 0.11	0.08 ± 0.09	0.14 ± 0.11	0.12 ± 0.12	0.11
Final foot contact (100%)	−0.02 ± 0.04	−0.01 ± 0.02	−0.04 ± 0.03	−0.04 ± 0.03	0.27
Normalized maximum knee moment (%)	0.76 ± 0.50^†^	0.71 ± 0.33	0.70 ± 0.28	0.68 ± 0.22	0.58

Values are mean ± SD. Significant difference: training ^*^, step dimension ^†^*p* < 0.05.

#### Joint Moments (Normalized)

Normalized ankle joint moments were affected by exercise (*F*_2,8_ = 11.3, *p* = 0.005; *d* = 0.46). Normalized ankle moment increased (pre, 0.89 ± 0.17; post, 1.39 ± 0.63), whereas knee joint moment decreased (*F*_2,8_ = 39.2, *p* = 0.0001; *d* = 0.75) (pre, 1.03 ± 0.38; post, 0.67 ± 0.26) after exercise training.

#### Negative Work

**FIGURE 2:**
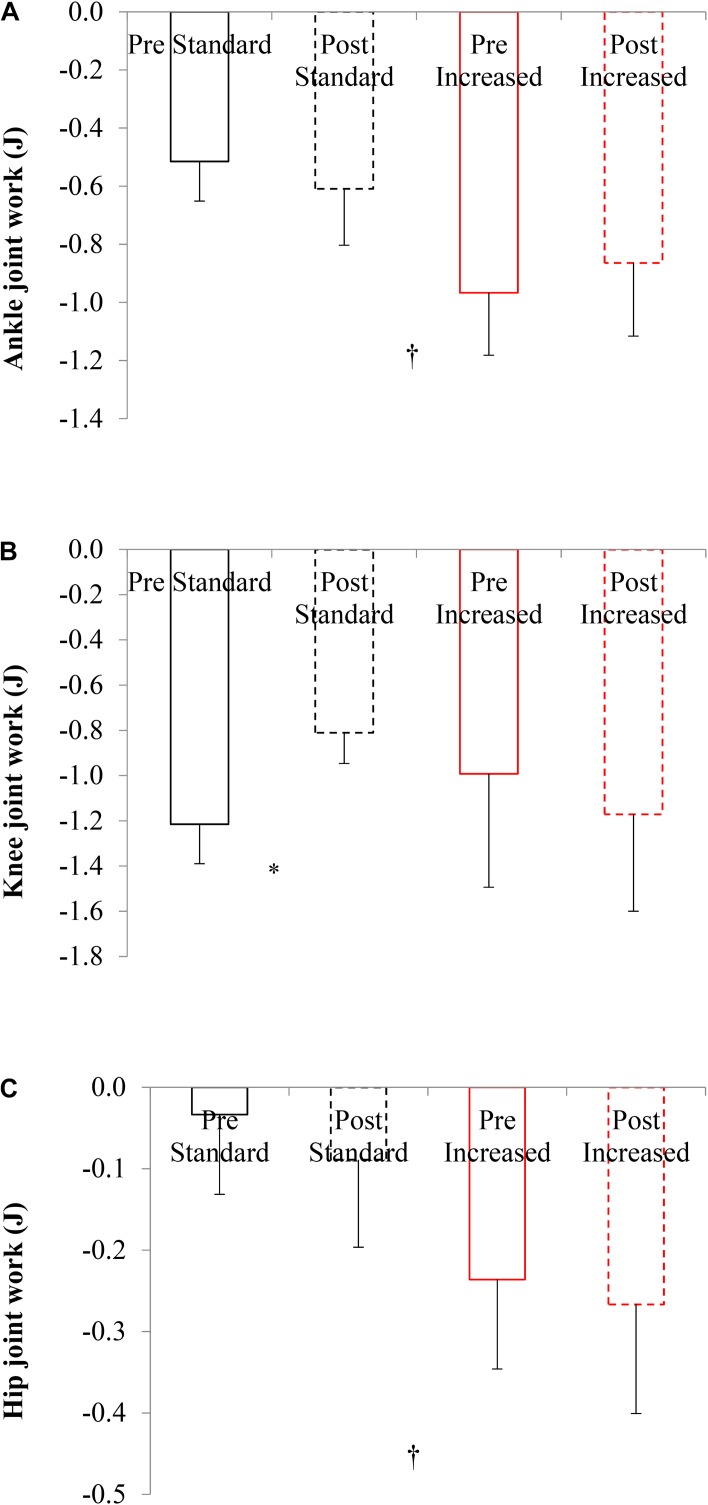
Joint work of older adults descending stairs of different dimensions before and after 16 weeks of exercise training for the ankle **(A)**, knee **(B)**, and hip **(C)** joints. Values are mean ± SD (*n* = 8); negative = eccentric work. Significant difference: training ^*^, step dimension ^†^*p* < 0.05.

Exercise training did not affect ankle or hip joint negative work when descending standard stairs (*p* > 0.48). Knee joint work on standard stairs decreased after training (*p* = 0.05; Figure 2). Total leg work decreased from before (−2.12 ± 0.11 J) to after training (−1.81 ± 0.13 J) when descending standard stairs (*p* = 0.03, *d* = 0.59).

#### Centre of Pressure (CoP)-Centre of Mass (CoM) Separation

**FIGURE 3:**
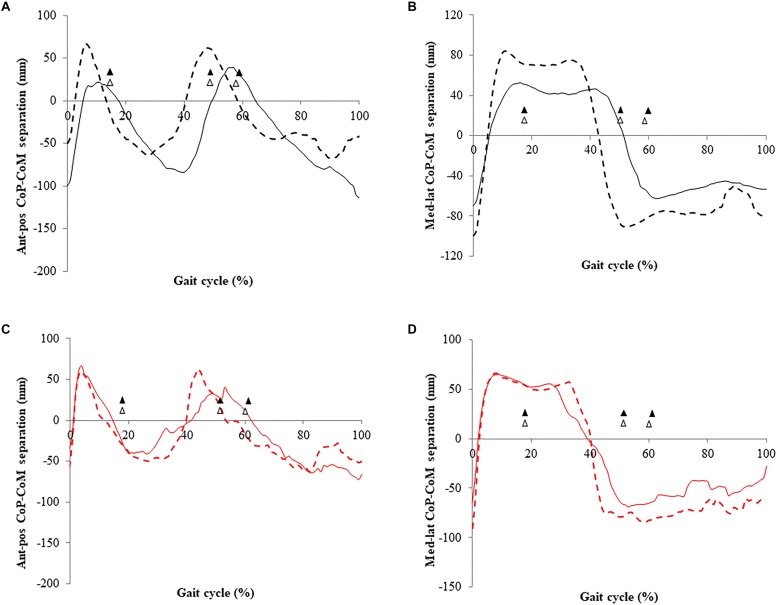
Center of pressure (CoP) and centre of mass (CoM) separation of older adults descending stairs of different dimensions before and after 16 weeks of exercise training. **(A)** Anterior-posterior separation (standard stairs); **(B)** medial-lateral separation (standard stairs); and **(C)** anterior-posterior separation (increased rise stairs); **(D)** medial-lateral separation (increased rise stairs). Gait cycle events (▲ standard, △ increased) refer to initial foot contact (0%), single-leg stance, double support, foot off, and final foot contact with the above step (100%); data correspond to the left limb. Values are mean (*n* = 8).

There was no difference in total excursion of the anterior-posterior CoP-CoM separation after exercise training (Figure 3 and Table 5). Peak CoP-CoM separation in posterior direction (CoM behind the CoP) (*F*_2,8_ = 11.75; *p* = 0.005; *d* = 0.5, difference 21 mm) increased after exercise training.

**TABLE 4:** Sagittal hip joint moments of older adults descending stairs of different dimensions before and after 16 weeks of exercise training.

	**Exercise group (*n* = 8)**		**Control group (*n* = 7)**		
			
**Gait cycle events**	**Pre**	**Post**	**Pre**	**Post**	***p*-value**
**Standard stairs**					
Initial foot contact (0%)	−0.23 ± 0.10	−0.16 ± 0.09	−0.15 ± 0.07	−0.17 ± 0.06	0.09
Single-leg stance	−0.13 ± 0.16	0.01 ± 0.21^*^	−0.20 ± 0.15	0.12 ± 0.17^*^	0.003
Double support	−0.18 ± 0.19	0.10 ± 0.21^*^	−0.19 ± 0.25	0.19 ± 0.20^*^	0.001
Foot off	−0.23 ± 0.11	−0.11 ± 0.18^*^	−0.25 ± 0.11	−0.14 ± 0.12^*^	0.01
Final foot contact (100%)	−0.11 ± 0.08	−0.09 ± 0.08	−0.11 ± 0.09	−0.06 ± 0.06	0.58
**Increased rise stairs**					
Initial foot contact (0%)	−0.20 ± 0.14	−0.19 ± 0.13	−0.17 ± 0.18	−0.10 ± 0.06	0.57
Single-leg stance	0.23 ± 0.32^†^	0.03 ± 0.25	0.14 ± 0.49	−0.07 ± 0.31	0.97
Double support	0.38 ± 0.28^†^	0.29 ± 0.32	0.30 ± 0.31	0.31 ± 0.23	0.31
Foot off	−0.17 ± 0.07^†^	−0.18 ± 0.10	−0.15 ± 0.08	−0.19 ± 0.11	0.36
Final foot contact (100%)	−0.06 ± 0.09	−0.08 ± 0.06	−0.05 ± 0.10	−0.08 ± 0.08	0.95

Values are mean ± SD. Significant difference: training ^*^, step dimension ^†^*p* < 0.05.

**TABLE 5:** Centre of pressure (CoP) and centre of mass (CoM) separation peaks of older adults when descending stairs of different dimensions before and after 16 weeks of exercise training.

	**Exercise group (*n* = 8)**		**Control group (*n* = 7)**		
			
**Anterior-posterior**	**Pre**	**Post**	**Pre**	**Post**	***p*-value**
**Standard stairs**					
**CoP-CoM separation (mm)**					
Total excursion	183.6 ± 32.7	169.0 ± 37.8	166.6 ± 23.5	167.2 ± 38.0	0.42
Maximum (positive): CoM behind CoP	58.2 ± 20.8	79.2 ± 33.5^*^	58.9 ± 25.0	70.7 ± 25.1	0.011
Minimum (negative): CoM in-front of CoP	−125.4 ± 17.6	−89.8 ± 21.0^*^	−108.0 ± 11.3	−96.2 ± 22.0	0.018
Medial-lateral					
Total excursion	143.6 ± 36.7	192.4 ± 37.1^*^	147.6 ± 16.7	163.4 ± 24.4	0.024
Maximum (positive): Right medial inclination	64.7 ± 17.6	85.4 ± 14.8^**^	63.9 ± 7.2	69.8 ± 12.4	0.017
Minimum (negative): Left medial inclination	−78.9 ± 26.6	−107.0 ± 23.2^**^	−83.3 ± 15.1	−93.7 ± 16.2	0.004
**Increased rise stairs**					
Total excursion	187.2 ± 20.0	174.1 ± 10.9	177.2 ± 19.0	177.8 ± 11.0	0.25
Maximum (positive): CoM behind CoP	94.5 ± 12.5	94.9 ± 13.8	94.8 ± 21.8	86.2 ± 11.9	0.10
Minimum (negative): CoM in front of CoP	−92.7 ± 32.0	−79.1 ± 16.2	−82.5 ± 15.0	−92.1 ± 14.8	0.13
**Medial-lateral**					
Total excursion	161.2 ± 19.8	172.0 ± 31.1	165.0 ± 42.7	160.2 ± 32.7	0.26
Maximum (positive): Right medial inclination	71.2 ± 13.1	75.5 ± 26.0	68.1 ± 25.0	69.2 ± 21.1	0.80
Minimum (negative): Left medial inclination	−90.0 ± 13.2	−96.5 ± 7.9	−97.0 ± 18.5	−91.0 ± 15.8	0.12

Values are mean ± SD (*n* = 8). Significant difference: training ^*^p < 0.05.

Total excursion of the medial-lateral CoP-CoM separation was greater after exercise training (*F*_2,8_ = 5.5; *p* = 0.04; *d* = 0.3, mean difference 48.8 mm).

#### Centre of Mass (CoM) Velocity and Acceleration

Exercise training did not affect the CoM velocity and CoM acceleration when descending standard stairs (Table 6).

**TABLE 6:** Velocity and acceleration of older adult’s center of mass (CoM) when descending stairs of different dimensions before and after 16 weeks of exercise training.

	**Exercise group (*n* = 8)**		**Control group (*n* = 7)**		
			
	**Pre**	**Post**	**Pre**	**Post**	***p*-value**
**Standard stairs**					
CoM peak velocity (m/s)	−0.43 ± 0.07	−0.45 ± 0.04	−0.49 ± 0.08	−0.50 ± 0.05	0.93
CoM peak accel. (m/s^2^) descent phase	−2.2 ± 0.6	−2.4 ± 0.4	−2.3 ± 0.7	−2.7 ± 0.7	0.58
CoM peak accel. (m/s^2^) landing phase	1.4 ± 0.5	1.7 ± 0.6	1.9 ± 0.6	1.8 ± 0.4	0.057
Peak foot velocity (m/s)	−1.39 ± 0.11	−1.36 ± 0.11	−1.54 ± 0.19	−1.61 ± 0.16	0.09
**Increased rise stairs**					
CoM peak velocity (m/s)	−0.58 ± 0.09	−0.55 ± 0.08	−0.64 ± 0.09	−0.66 ± 0.14	0.17
CoM peak accel. (m/s^2^) descent phase	−3.3 ± 0.7	−3.3 ± 0.8	−3.7 ± 1.1	−4.4 ± 1.1^*^	0.04
CoM peak accel. (m/s^2^) landing phase	2.1 ± 0.4	2.1 ± 0.2	2.3 ± 0.6	2.5 ± 0.7	0.34
Peak foot velocity (m/s)	−1.64 ± 0.21	−1.51 ± 0.26	−1.81 ± 0.19	−1.76 ± 0.25	0.54

Values are mean ± SD (*n* = 8). Significant difference: training ^*^p < 0.05.

### Increased Rise Stair Descent

#### Temporal-Spatial Characteristics

Step rise height did affect gait characteristics during stair descent with increased rise stairs, requiring lower stride frequency (67.9 ± 9.6 steps/min, *p* = 0.001), longer step length (0.40 ± 0.04 m, *p* = 0.001) and prolonged single support (57.3 ± 8.3%, *p* = 0.001), but similar double support (20.7 ± 8.2%, *p* > 0.05). Exercise training reduced stride frequency when descending increased rise stairs (61.0 ± 13.5 steps/min, *p* = 0.04).

#### Joint Moments (Absolute)

When compared to standard stairs descending increased rise stairs required greater ankle, knee and hip joint moments (*p* < 0.001; Tables 2–4), greater knee flexion moments during both lowering and landing phases (*p* = 0.004), but reduced knee extension moments during landing (*p* < 0.001).

Peak ankle joint moments during landing showed a tendency to decrease after exercise training (*p* = 0.06; *d* = 0.46), whereas peak knee joint extension moments during lowering increased after exercise training (*p* = 0.06; *d* = 0.37). Hip joint moments were unaffected by exercise training.

#### Joint Moments (Normalized)

Normalized ankle joint moments were affected by stair dimension (*F*_2,8_ = 11.3, *p* = 0.005; *d* = 0.46). Normalized ankle and knee joint moments were greater when descending increased rise stairs (*p* < 0.001; Tables 2, 3), when compared to standard stairs.

Normalized ankle and knee joint moments when descending increased rise stairs were unaffected by exercise training (*p* > 0.4).

#### Negative Work

Descending increased rise stairs required greater ankle (rise effect, *p* = 0.0001) and hip joint work (rise effect, *p* = 0.0001), when compared to standard stair descent. In addition, total leg work was greater descending increased rise stairs (−2.58 ± 0.35 J, *p* = 0.02; *d* = 0.34), when compared to descending standard stairs (−2.09 ± 0.16 J).

Exercise training had no effect on the amount of negative joint work at the ankle, knee or hip joint, when descending increased rise stairs (*p* > 0.48).

#### Centre of Pressure (CoP)-Centre of Mass (CoM) Separation

Descending stairs of increased rise involved greater CoP-CoM separation in anterior-posterior (*F*_2,8_ = 7.0; *p* = 0.02; *d* = 0.35) and medial-lateral directions (*F*_2,8_ = 23.43; *p* = 0.0001; *d* = 0.64), when compared to standard stairs (Figures 3C,D).

Increased rise stair descent involved greater peak separation in the posterior direction (CoM behind CoP) (*F*_2,8_ = 24.1; *p* = 0.0001; *d* = 0.67) and lesser separation in the anterior direction (CoM in-front of CoP) (*F*_2,8_ = 14.23; *p* = 0.002; *d* = 0.52), when compared to standard stair descent.

Exercise training did not affect the total excursion of the CoP-CoM separation in anterior-posterior and medial-lateral directions (*p* > 0.02) when descending increased rise stairs (Figure 3).

#### Centre of Mass (CoM) Peak Velocity and Acceleration

Acceleration (rise effect, *p* = 0.0001; time effect, *p* = 0.003), and lead foot velocity (rise effect, *p* = 0.002) (*p* < 0.002; Table 6), when compared to descending standard stairs.

Exercise training did not affect the CoM velocity and CoM acceleration when descending stairs of increased rise (*p* > 0.1). However, the control group’s CoM peak downward acceleration was slightly greater in the descent phase (from −3.7 m/s^2^ to −4.4 m/s^2^; *p* = 0.04; *d* = 0.28) on increased rise stairs after 16 weeks of not training.

## Discussion

The study describes the lower-limb joint kinetics and the CoM motion of older adults descending stairs of different step dimensions before, and after 16 weeks of resistance exercise training. The exercise training, which included plantarflexor stretching, improved ankle torque (at 60°⋅s^–1^) and knee torque (from 60 to 240°⋅s^–1^), and maximum dorsiflexion joint range when assessed by isokinetic dynamometry. Importantly, exercise had a positive effect of redistributing joint moments in standard rise stair descent, specifically by allowing a greater ankle and hip moment, whilst reducing knee moments. This redistribution of joint moments after exercise was linked with a balance control strategy that could be regarded as safer, by maintaining the CoM further behind the CoP during stair descent. Increasing stair rise by 50% required the participants to take longer steps. This resulted in prolonged single-limb support, increased lower-limb joint moments, increased downward acceleration of the CoM and presented further challenge to (anterior-posterior) postural stability, when compared to standard stair descent. Exercise training could not overcome these additional biomechanical demands when descending stairs in the common step-over-step manner. To our knowledge, this is the first demonstration in older adults that exercise training can positively affect stepping biomechanics when walking down stairs.

### Effects of Exercise Training

Exercise training enabled older adults to use their ankle plantarflexors more and their knee extensors less, in standard rise stair descent. Age-related deteriorations in plantarflexor biomechanics exceed those of other muscle groups (Lark et al., 2003; Silder et al., 2008; Reeves et al., 2009), yet our training intervention increased maximum eccentric ankle joint moment (normalized to body mass) and dorsiflexion angle by 35 and 11%, respectively, when assessed by isokinetic dynamometry. During stair descent the absolute ankle joint moment increased after training, peaking with the left, load-bearing foot in single-leg support (at maximum dorsiflexion angle), when stepping down with the right foot; and in the trailing left foot after double support, to negotiate the load-bearing step. The latter phase corresponded to the second ankle, and then knee joint moment peak, and involved a large sagittal plane separation between the CoM and CoP indicating anterior lean (CoM in front of CoP), and subsequently posterior lean (CoM behind CoP) from the left foot off.

After exercise training, there was greater posterior leaning (i.e., the CoM distributed further behind the application of CoP) to control lowering of body mass in single-support when stepping down (Figure 3A). This may reflect a safer balance control strategy through positioning of CoM further behind the CoP after exercise training, reducing the risk of falling forward during stair descent. The compromise, however, is that this strategy requires higher joint moments with the ground reaction force being applied more posteriorly, which would consequently generate a larger external moment arm, but this was enabled through the increased joint moment capability provided by exercise training. Increased hip joint flexion moment in this early-swing phase is unlikely to have contributed to these differences in balance control (Simonsen et al., 2012). Exercise training, particularly leg-press and calf-press contractions, strengthened ankle and hip musculature, and enabled participants to tolerate the development of higher joint moments and adopt a more posterior CoM displacement. This joint moment redistribution was accommodated by reduced knee joint moment in a phase of high demand (swinging the left leg to step down), as the maximum knee capabilities measured by dynamometry also improved.

We combined resistance exercise with ankle stretching for the following two reasons. Firstly, limitation of mobility of the ankle and knee joints is prevalent in aging, and these in turn, may impair dynamic stability when stepping down in stair descent (Bosse et al., 2012). Secondly, unlike the knee joint (Andriacchi et al., 1980; Mian et al., 2007), the ankle approaches it’s maximum dorsiflexion joint range (∼20–30°) (Protopapadaki et al., 2007; Reeves et al., 2008a), and moment limits (∼75%) when descending stairs, and so has less reserve capacity than the knee joint. Thirdly, in combination these present high falls risk, due to limited ankle motion predisposing to a “controlled fall,” and limited ankle force development comprising the individual’s ability to respond to unexpected perturbations when stepping down.

Training-induced strength changes may have increased the reliance upon ankle joint moment to sustain single-leg support in stair descent. Redistribution of lower-limb joint moments occurred at a phase of high demand in stair descent. In particular, the capability for the ankle joint to operate beyond maximum capacity at extreme dorsiflexion joint range. The maximum eccentric joint moment was greater after exercise training by 30 to 43% at the knee (angular velocity-specific), and by 35% at the ankle (Table 1). When joint moments in stair descent were normalized to maximum eccentric moments quantified by dynamometry, the knee exceeded (103%) and ankle neared (89%) maximum strength capabilities. After exercise training, the knee operated at lower (67%), and the ankle at higher (139%) proportion of maximum (Tables 2, 3). This may seem paradoxical, however, during standard stair descent the gastrocnemius would have been contracting in a bi-articular manner, acting across the ankle and knee joints. Whilst during isokinetic dynamometry testing, the gastrocnemius would have been contracting in an isolated, uni-articular manner, and thus producing lower summated force. Similarly, Samuel et al. (2011) reported knee extensor moments in stair descent exceeding maximum isometric moments by 120% for adults aged over 60 years.

The excessive task demands of stair descent leave minimal reserve, with which older adults can capitalize on in unexpected situations. Mian et al. (2007) combined aerobic and resistance exercise training for older adults over 12 months, without demonstrating change in gait speed and kinematics in stair descent. We progressed on this intervention by incorporating stretching and rate of torque development exercises, specifically for the ankle joint; that which is the limiting-factor at the most demanding point of stair descent (i.e., single-leg support). Additionally, in quantifying gait kinetics and movement strategies we have demonstrated that 16 weeks of exercise training can lead to older adults descending stairs with redistributed joint moments and altered postural stability.

During descent, the joint work was negative, as energy was absorbed. Before exercise training the knee joint performed the most negative work (−1.22 J), when compared to the ankle (−0.52 J), and hip (−0.03 J) joints. However, negative work by the knee joint significantly reduced after exercise training (−0.81 J), whilst remaining similar at the ankle and hip joints (Figure 2). This may signify that less energy absorption (in eccentric quadriceps contraction) was required in decelerating the knee joint when stepping downward. Theoretically this would require the older adults to generate a narrower impulse (i.e., applying higher torque more rapidly to adequately decelerate body mass). It appears after exercise training the lower-limb joints share energy absorption through the swing phase (kinetic energy production) to stepping down (dissipation). This has relevance for stair safety and potentially injury, as stair descent requires the knee joint to absorb 3.8 times more maximum power, than level walking (Riener et al., 2002). The present positive results of exercise training on stepping biomechanics in older people executing the standard rise descent task are particularly relevant for reducing the risk for stair falls, as most of the private and public staircases encountered daily are regulated, with a rise around the 170 mm value examined here. Interventions to improve stair safety in older people should therefore consider improving not only the environment (Jacobs, 2016), but also the individual’s functional capacities.

### Effects of Increasing Riser Height

The second part of this study involved investigating whether descending stairs of increased riser height would impact on the biomechanical strategies adopted by older adults, and subsequently, whether 16 weeks of exercise training could further affect their biomechanics. Our experimental staircase was configured to closely replicate the ranges of stair riser height negotiated in daily life, that is, standard stairs (170 mm) and increased rise stairs (255 mm). Standard stair descent provided an experimental benchmark, with which to compare the biomechanical demands to increased rise stair descent. Increased rise stair descent represented a much higher task demand that may be encountered in certain circumstances, for example, in steps on public transport (Institute for Transportation, and Development Policy [ITDP], 2018), pre-1950s residential dwellings and unregulated staircases (HM Government, 2013). Descending stairs with a rise of 255 mm is demanding for the young, particularly at the ankle and knee joints (Spanjaard et al., 2008). Adopting the common step-over-step strategy in the present study, a 255 mm rise required the older participants to increase step length (14.3%), reduce stride frequency (−17.7%) and prolong single-leg support (24.6%) in lowering greater vertical distance to the below step. As in the young (Riener et al., 2002), descending stairs of increased rise involved higher joint moments at the ankle, knee and hip in the old. The point of highest demand was in single-leg support, which involved peak dorsiflexion and high knee extension moments. From left foot contact in forward continuance, the knee flexion joint moment was greater on increased rise stairs until the right swing phase to the step below. This coincided with lower knee extension moment, when compared to standard rise stairs.

Whereas standard stair descent was most demanding on the knee joint, by increasing riser height the greatest demand was placed on the ankle joint. According to dynamometry-normalized joint moments, at the point of highest demand the ankle and knee joints were operating at 89 and 103% of maximum capacity in stair descent, respectively. Conversely, descending increased rise stairs required the ankle joint to work at 175%, and the knee joint 76% of maximum moment capacity at the point of highest demand. As discussed above, the ankle operating beyond maximal capacities after training may be attributed to a gastrocnemius bi-articular action for (dynamic) stair descent, and uni-articular action for (static) isokinetic dynamometry testing.

No falls or events of postural instability occurred in stair descent in our cohort, indicating that these community-dwelling older adults were capable of coping with excessive demand at the ankle joint. However, for adults with functional limitations at the ankle it would be extremely difficult to safely descend stairs of increased rise unaided. Where environmental aids are not present, older adults can adopt non-cyclical gait strategies (e.g., step-by-step or side-stepping) to control gait speed, without prolonging single-leg support in stair descent. For example, side-stepping can be used to reduce ankle plantarflexor and hip extensor demand, without enlarging the lead limb joint moments upon step contact (King et al., 2018). These compensatory strategies, however, involve the preferential use of a single lead limb, which may be unsuitable to recover bilateral function in individuals with unilateral muscle (Metcalfe et al., 2013).

For standard stairs, exercise training was effective in alleviating the mechanical knee joint work in descent. From an interventional perspective, mechanical joint work was more heavily influenced by stair dimension, than exercise training. Increased rise stairs required greater negative work at the ankle (−0.97 J) and hip (−0.24 J) joints in descent, when compared to standard stairs (Figure 2). Riener et al. (2002) found similar involvement for increased joint power maximums at the ankle (67.3%) and hip (24.3%) when increasing stair inclination during descent. Stair descent involves large dorsiflexion joint range and support moments; if an individual lacks ankle joint range and/or functional strength when descending increased rise stairs, the lowering of mass to the below step will be greatly compromised. For this reason, our older cohort underwent lower limb resistance exercise, with rate of torque development and stretching specifically for the ankle plantarflexors, with which to enhance maximum dorsiflexion motion and support moments in the lowering phase. Thus, an important step in developing future exercise-based interventions would be to prioritize the ankle joint, particularly targeting plantarflexor muscular capacities for older adults in an ecologically valid environment.

The altered gait pattern required to safely descend increased rise stairs not only imposed greater functional demand, but also challenged movement control. Descending stairs of increased rise resulted in higher CoM and lead foot velocities, and CoM acceleration toward the ground, when lowering to the below step. The descriptive statistics indicate greater variability in CoM motion (see Table 6) when descending increased rise stairs. This is reasonable considering the greater vertical distance that must be overcome when stepping downward with increased rise steps. It is equally plausible that this added demand also contributed to the slightly faster CoM peak downward acceleration (from −3.7 m/s^2^ to −4.4 m/s^2^; *d* = 0.28) in the control group when descending increased rise stairs, after 16 weeks of no training. The speed at which CoM downward velocity is arrested on landing in stair descent is mediated by the ankle joint’s capability to produce eccentric torque at high angular velocities (Buckley et al., 2013). With older adults already operating at near maximum ankle capacity for standard stairs, and in-excess of maximum for increased rise stairs, individuals would have to manipulate their movement strategy. This was characterized by a reduced CoP-CoM displacement in the frontal plane (i.e., medial-lateral direction), and larger CoP-CoM displacement in the sagittal plane (i.e., anterior-posterior) during increased rise stair descent.

Descending increased rise steps (255 mm) requires greater plantarflexor and dorsiflexor activity upon landing (Gerstle et al., 2018), which may reflect a challenge to medial-lateral stability. Increased maximal ankle capacities following exercise training may have contributed to assist in controlling medial-lateral CoM stability. Progressing forward into single-leg support with the right foot leaving the above, increased rise step, the CoM was positioned further behind the CoP of the left foot, indicating greater posterior lean in descent (Figures 3A,C). Thereafter, from the right swing phase into double support the CoM was positioned closer to the CoP, indicating reduced anterior lean. No previous study has reported functional demand and balance control at the ankle, knee, and hip joints during increased rise stair descent for healthy, older adults. Greater trunk posterior leaning when lowering body mass further on to the below step, and lesser anterior leaning, upon the step contact (and mass acceptance) can be seen as a safe strategy in preventing uncontrolled CoM acceleration.

### Effects of Exercise Training at Increased Riser Height

Older adults adopt cautious movement strategies in stair descent to safely maintain lowering of mass, including prolonging the trail limb muscle co-contraction at the ankle and knee (Buckley et al., 2013) and reducing lead foot heel clearance (Kunzler et al., 2017). By increasing the riser height, and therefore task demand, our healthy older adults adopted alternative movement strategies to control safe stair descent. However, 16 weeks of exercise training was ineffective in enabling participants to further cope with the additional demands of increased rise stair descent. Our increased riser steps (255 mm), were only 15% higher than the maximum rise recommended for new private domestic stairs (220 mm) (HM Government, 2013). Community-dwelling older adults can be expected to negotiate similar riser heights in private and public buildings constructed prior to new national regulations.

The knee joint produced greater moment in single-leg support on increased rise stairs after exercise training. However, when normalized to dynamometry-assessed maximum eccentric moments, the exercise training had no effect on lower-limb joint moments in stair descent. Similarly, the amount of negative work performed by a lower-limb joint was constrained by stair rise, and not influenced by exercise training. Training improvements in ankle torque production may have been sufficient to support the contralateral limb, as body mass was lowered to the step below, but for increased rise stairs, it appears ankle adaptations could not overcome the additional demand of lowering greater vertical distance. In this case, greater knee extension moments when descending increased rise stairs following training may have compensated, particularly to counter large knee flexion angles, which exceed those of standard rise stairs (Protopapadaki et al., 2007).

In the present study, participants descended each stair step-over-step, at a self-selected pace. Therefore, by necessity the descent tasks were different between stair configurations, but theoretically identical before and after training. However, as stride frequency lowered for increased rise stair descent after training, the same motor task was not being performed. Whether this reflects a more stable and controlled gait is unclear as movement control remained unchanged after exercise. Unsurprisingly, exercise training did not affect balance control (i.e., CoP-CoM separation) when descending increased rise stairs. The greater downward distance to the below step required not only an altered movement strategy to control CoM velocity, but also the ankle joint to operate beyond functional capacity. The increased rise was too high to “allow” any adaptation after exercise training. This is also supported by the downward CoM velocity and acceleration, which remained unchanged. The improvements in maximum functional capabilities at the ankle, knee and hip were nullified by the requirement to further control CoM acceleration and to generate sufficient external joint moments with which to alter postural strategies (i.e., CoP-CoM separation). Our older cohort was instructed to descend stairs using the step-over-step gait pattern, and therefore participants could not adapt their strategy to reduce the biomechanical demand. This further indicates that the increased rise stairs posed maximal demand, for which exercise training could not overcome, as opposed to older adults having to adopt differential gait strategies (e.g., side-stepping) to cope with increase rise descent.

## Conclusion

This study demonstrated that combined, lower-limb resistance and stretching exercise training can confer functional improvements in older adults when descending a staircase with a standard 170 mm riser height. Specifically, enabling our older cohort to redistribute lower-limb joint moments and adopt movement strategies to cope with the task demands of stair descent, including a safer balance control strategy. However, exercise training could not overcome the extra biomechanical demands imposed by increasing riser height by 50%. The post-exercise improvements at 16 weeks in the standard riser descent task highlights the relevance of incorporating appropriate exercise training in interventions aiming at improving daily stair safety in older people.

## Ethics Statement

This study was carried out in accordance with the recommendations of the Ethics and Governance guidelines of the Manchester Metropolitan University, with written informed consent from all participants. All participants provided written informed consent in accordance with the Declaration of Helsinki. The protocol was approved by the ethics committee of the Manchester Metropolitan University, United Kingdom. We can confirm no vulnerable populations were involved as participants in the study.

## Author Contributions

NR, CM, JB, VB, DJ, and MR contributed to the conception and design of the study. NR performed the data collection. JG performed the subsequent data and statistical analyses. JG, NR, CM, JB, VB, and MR interpreted the results of the research. JG wrote the first draft of the manuscript. NR, CM, JB, VB, and MR reviewed the manuscript, as JG revised the subsequent manuscript versions. All authors contributed to the final manuscript revision, and read and approved the submitted version.

## Conflict of Interest Statement

MR was employed by the company Building Research Establishment (BRE) during the research study. MR is currently employed by the company Rise and Going Consultancy. The BRE’s goal is “to make the built environment better for all,” and the company supports independent research by allowing researchers and engineers collaborate together in creating evidence-based solutions. The remaining authors declare that the research was conducted in the absence of any commercial or financial relationships that could be construed as a potential conflict of interest.
